# Ultrasmall Surface-Charge-Modified Tantalum Oxide Nanoparticles for the Assessment of Articular Cartilage Using Contrast-Enhanced Computed Tomography

**DOI:** 10.1021/acsnano.5c15722

**Published:** 2026-01-14

**Authors:** Jiri Jäntti, Juuso Tuppurainen, Anisha Joenathan, Henri P. P. Leskinen, Annunzia M. Cagnoni, Heta Mertano, Milka Poimala, Ervin Nippolainen, Isaac O. Afara, Juuso T. J. Honkanen, Hanna Matikka, Juha Töyräs, Brian D. Snyder, Kathryn S. Stok, Brad B. Nelson, Mark W. Grinstaff, Janne T. A. Mäkelä

**Affiliations:** † Department of Technical Physics, University of Eastern Finland, Kuopio 70211, Finland; ‡ Diagnostic Imaging Center, Kuopio University Hospital, Wellbeing Services County of North Savo, Kuopio 70210, Finland; % Medical Physics, Mikkeli Central Hospital, South Savo Wellbeing Services County, Mikkeli 50100, Finland; § Division of Materials Science, 1846 Boston University, Boston, Massachusetts 02215, United States; ⊥ Department of Biomedical Engineering, The University of Melbourne, Melbourne 3010, Australia; ∥ Rehabilitation Sciences Institute, University of Toronto, Toronto, Ontario M5G 1V7, Canada; ¶ Joint Department of Medical Imaging, University Health Network, Toronto, Ontario M5G 2M9, Canada; # School of Electrical Engineering and Computer Science, The University of Queensland, Brisbane 4072, Australia; ∇ Center of Oncology, Kuopio University Hospital, Wellbeing Services County of North Savo, Kuopio 70210, Finland; ○ ICMT and Development Services, Kuopio University Hospital, Wellbeing Services County of North Savo, Kuopio 70210, Finland; @ Science Service Center, Kuopio University Hospital, Wellbeing Services County of North Savo, Kuopio 70210, Finland; ◆ Department of Orthopedic Surgery, Boston Children’s Hospital, Boston, Massachussets 02115, United States; $ Orthopaedic Research Center, C. Wayne McIlwraith Translational Medicine Institute, Colorado State University, Fort Collins, Colorado 80523, United States; & Departments of Biomedical Engineering and Chemistry, Boston University, Boston, Massachussets 02215, United States

**Keywords:** cartilage imaging, microcomputed tomography, contrast agent, nanoparticles, osteoarthritis, lesions

## Abstract

The size and surface properties of nanoparticles (NPs) define their interactions with biological tissues and impact their effectiveness for targeted imaging and therapeutic applications. Here, we investigate ultrasmall tantalum oxide nanoparticles (Ta_2_O_5_-NPs) as computed tomography (CT) contrast agents and the effects of NP surface charge on diffusion in *ex vivo* human cartilage samples, as well as after intra-articular administration in intact rabbit and equine joints. Controlled hydrolysis of tantalum­(V) ethoxide, followed by coating with positively charged trimethylammonium and nonionic poly­(ethylene glycol) (PEG) ligands at different ratios, affords the approximately 3 nm positively charged (Ta_2_O_5_-cNPs) and neutral (Ta_2_O_5_-nNPs) tantalum oxide nanoparticles. Ta_2_O_5_-cNPs readily diffuse into human cartilage as measured by microcomputed tomography, and the Ta_2_O_5_-cNPs partition positively correlates with key indicators of early-stage osteoarthritis to include the proteoglycan content, equilibrium Young’s modulus, and porosity (stress-relaxation time constant) while inversely with viscosity (phase shift). In contrast, the neutral Ta_2_O_5_-nNPs reside at the cartilage surface, triple the attenuation difference at the cartilage–fluid boundary, accumulate within microscopic surface injuries, and enable clear detection of surface injuries and lesions. When extended to intact *ex vivo* animal models, Ta_2_O_5_-cNPs diffuse into rabbit knee cartilage over 24 h, while Ta_2_O_5_-nNPs delineate fissures and partial erosions in equine carpal cartilage. In conclusion, customizing the Ta_2_O_5_-NPs surface charge allows, or prevents, their diffusion into cartilage, enabling distinct CT imaging applications: diffusible Ta_2_O_5_-cNPs assess the cartilage structure and function, while nondiffusible Ta_2_O_5_-nNPs enhance cartilage segmentation and detect lesions in both human *in vitro* and animal *ex vivo* models.

## Graphical abstract

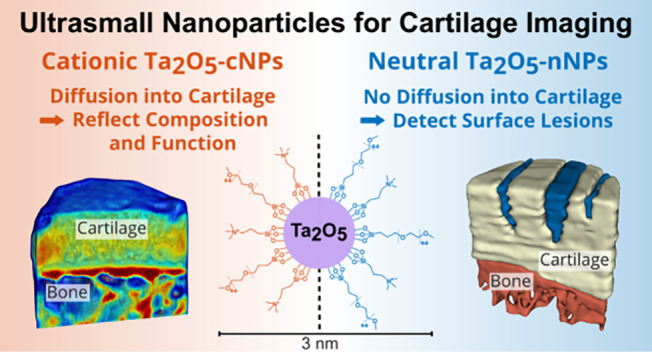

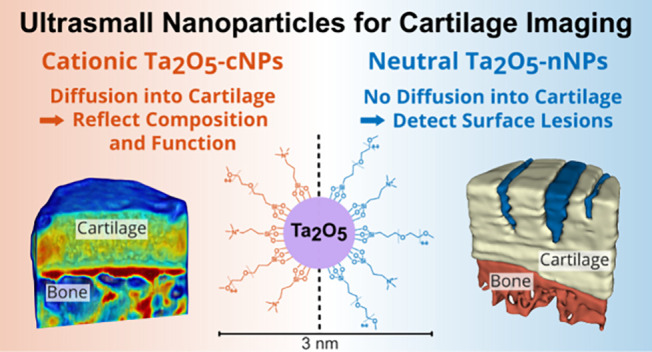

Detection of the early stages of osteoarthritis (OA) rarely occurs due to mild symptoms limiting opportunities for early intervention and effective disease management.^1^ In contrast, traumatic events such as sports injuries, vehicle accidents, or falls act as early indicators of post-traumatic OA, prompting medical evaluation and follow-ups, and, thus, a greater likelihood of detection.^2^ However, the microscopic cartilage changes in plain radiography may be negligible and remain undiagnosed in primary care for years until they further manifest.^3^ Advanced imaging technologies that reveal the cues of early degeneration are of significant interest.

The microscopic cartilage changes in early-stage OA include proteoglycan (PG) loss and collagen network fibrillation and at the later stage collagen loss, which leads to impairment in the biomechanical properties, e.g., equilibrium and instantaneous stiffness as well as increased permeability of the tissue.^4^ Contrast-enhanced computed tomography (CECT) imaging enables assessment of these changes.^5,6^ In CECT imaging, contrast agents improve the visibility of cartilage from the synovial fluid or, upon penetration of the cartilage zones, reflect its structural and functional properties. Traditionally, negatively charged iodinated (*k*-edge = 33.2 keV) small-molecule contrast agents have been widely utilized in clinical settings due to their biocompatibility and established use.^7–9^ However, their negligible penetration into cartilage, which has a negative fixed charge density, limits their effectiveness.^8–10^ Cationic small-molecule contrast agents (e.g., CA4+ and cationic bismuth contrast agent) offer significant advancement because their strong affinity for negatively charged PGs improves specificity and cartilage visibility.^6,11–14^ These cationic contrast agents reveal changes in critical OA markers including the PG^6,13,15,16^ and water contents,^17^ collagen distribution,^17,18^ and equilibrium modulus,^5^ thereby facilitating the detection of OA.

Nanoparticles (NPs) are widely utilized in biomedical imaging applications, for example, in cancer imaging,^19,20^ atherosclerotic plaques imaging and monitoring drug delivery to plaques,^21,22^ thrombi detection in stroke,^23^ and lung imaging and diagnosing intra-alveolar inflammation.^24,25^ Various imaging modalities such as fluorescence imaging, computed tomography (CT), magnetic resonance imaging, positron emission tomography, single-emission computed tomography, and ultrasound imaging capitalize on the use of NP contrast agents.^26,27^ The appealing properties of NP contrast agents for CECT imaging include a higher proportion of the heavily attenuating element in their structure compared to molecular contrast agents, and, more importantly, a core structure and surface functionality that is customizable for different applications.^28–31^

Particularly in CT imaging of cartilage, only a few NP contrast agents are introduced, likely because the NP size must be extremely small to diffuse into cartilage. Large NPs, however, still provide some benefits; for example, we have developed bismuth NPs (*k*-edge = 90.5 keV; *d* < 200 nm) that are heavily X-ray-attenuating, which highlight the synovial space and enable segmentation by delineating the fluid–cartilage interface.^32,33^ We have also pioneered the use of ultrasmall tantalum oxide nanoparticles (Ta_2_O_5_-NPs; *k*-edge of 67.4 keV and diameters of 2.5–6 nm), which diffuse into the cartilage matrix.^34–36^ The versatility of Ta_2_O_5_-NPs arises from their tunable surface coatings, allowing for anionic, cationic (Ta_2_O_5_-cNPs), or neutral (Ta_2_O_5_-nNPs) coatings. Ta_2_O_5_-cNPs, in particular, enable imaging of PG distribution and show promise for 3D histology of cartilage.^34–36^ Beyond their ability to directly reflect the PG content and indirectly reflect the biomechanical properties (e.g., equilibrium and dynamic stiffness), their diffusion depends on the collagen structure, fibrillar and nonfibrillar functionality, and cartilage permeability because their relatively larger size distinguishes them from molecular contrast agents like CA4+.^36,37^

Here, we prepare ultrasmall (∼3 nm) Ta_2_O_5_-NPs with identical tantalum oxide cores but alter the ratio of positively charged trimethylammonium and nonionic poly­(ethylene glycol) (PEG) ligands on their surfaces. This NP design allows us to isolate and test how the surface charge influences NP diffusion, cartilage targeting, and cytocompatibility at a size scale that is still capable of penetrating the cartilage matrix. To our knowledge, this is the first direct comparison of cationic versus neutral ultrasmall Ta_2_O_5_-NPs of otherwise identical size and composition. We hypothesize that the surface charge critically governs NP diffusion in cartilage and that the PG content modulates this interaction. To test this, we combine NP physicochemical characterization with cytotoxicity assessment, cartilage biomechanical and compositional analysis, and CECT imaging in human cartilage plugs, complemented by translational *ex vivo* joint-level validation in rabbit and equine models.

## Results

The hydrodynamic diameters of Ta_2_O_5_-cNPs and Ta_2_O_5_-nNPs were 2.77 nm [95% confidence interval (CI) 1.86, 3.68] and 3.08 nm (95% CI 1.53, 4.63), respectively. The ζ potential was 25.63 mV (95% CI 22.26, 29.00) for Ta_2_O_5_-cNPs and −0.98 mV (95% CI −3.80, 1.84) for Ta_2_O_5_-nNPs. The very small negative charge likely arises due to the negative Ta_2_O_5_ core. The core sizes of Ta_2_O_5_-cNPs and Ta_2_O_5_-nNPs were 1.1 nm (95% CI 1.0, 1.2) and 1.2 nm (95% CI 1.1, 1.2), respectively, determined by ultrahigh-resolution analytical scanning transmission electron microscopy (UHR-STEM). Fourier transform infrared (FTIR) spectra of Ta_2_O_5_-cNPs and Ta_2_O_5_-nNPs exhibited characteristic peaks, consistent with previous studies,^34,35^ confirming successful ligand attachment to the NP surface (Figure 1). The cytotoxicity of Ta_2_O_5_-nNPs has been reported previously, with a half-maximal inhibitory concentration (IC_50_) value of 20.07 mg/mL.^35^ In this study, Alamar Blue assay showed an IC_50_ value of 22.42 mg/mL for Ta_2_O_5_-cNPs (Figure S1).

**Figure 1:**
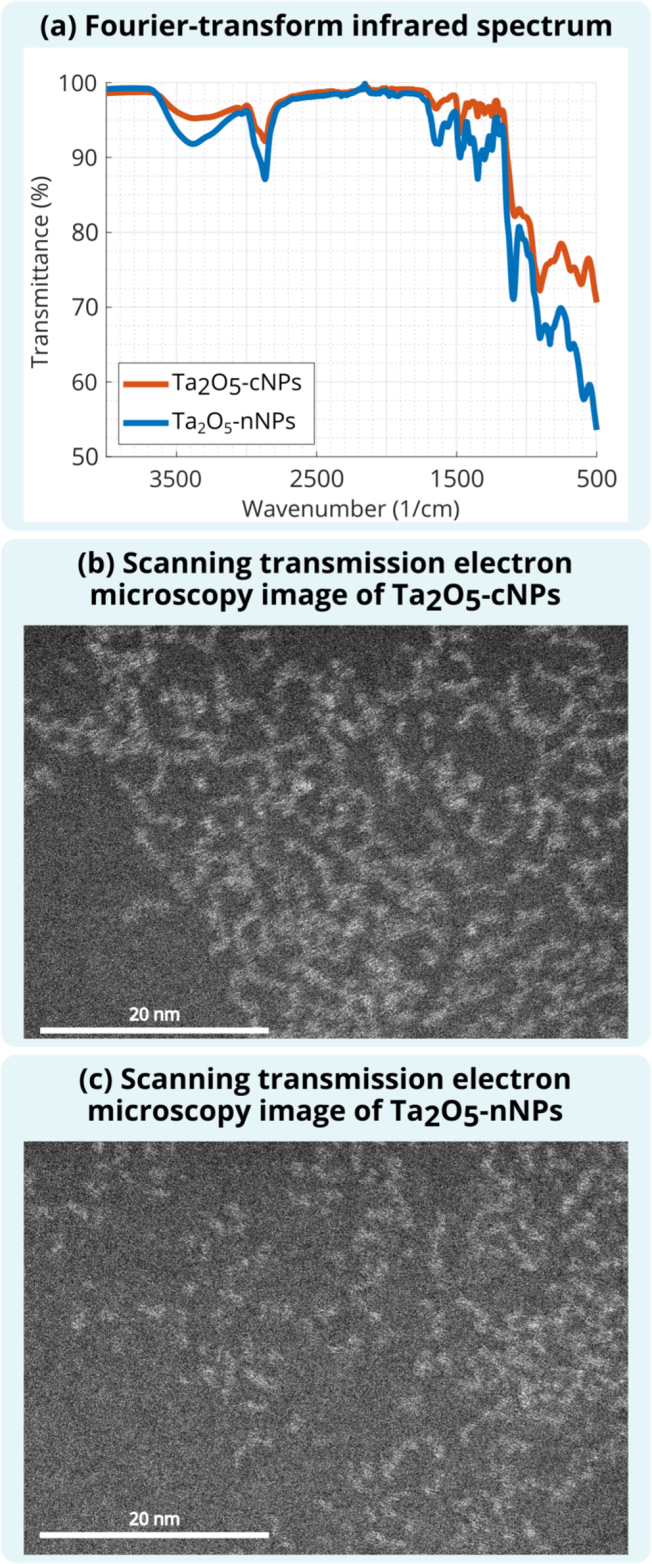
(a) Infrared light transmittance at different wavenumbers measured by using FTIR spectroscopy confirmed silane ligand conjugation to the NPs. Dark-field UHR-STEM images of (b) Ta_2_O_5_-cNPs and (c) Ta_2_O_5_-nNPs showing that the NP core size was 1.1 nm (95% CI 1.0, 1.2) and 1.2 nm (95% CI 1.1, 1.2), respectively. Bright areas show the cores of the NPs.

Ta_2_O_5_-cNPs diffused into human cartilage, reaching a partition value of 39.25% (95% CI 38.33, 40.18) in 72 h, whereas Ta_2_O_5_-nNPs showed no diffusion, with a partition value of −3.65% (95% CI −6.86, −0.43) at the same time point (Figure 2). The maximum partition (*P*_max_) of Ta_2_O_5_-cNPs was 56.49% (95% CI 46.48, 66.51), and the diffusion time constant (τ) value was 62.85 h (95% CI 41.63, 84.06). On the basis of the observed lack of diffusion of Ta_2_O_5_-nNPs, we further investigated whether intentionally induced cartilage surface lesions would allow for their diffusion or enhance their utility in revealing these lesions.

**Figure 2:**
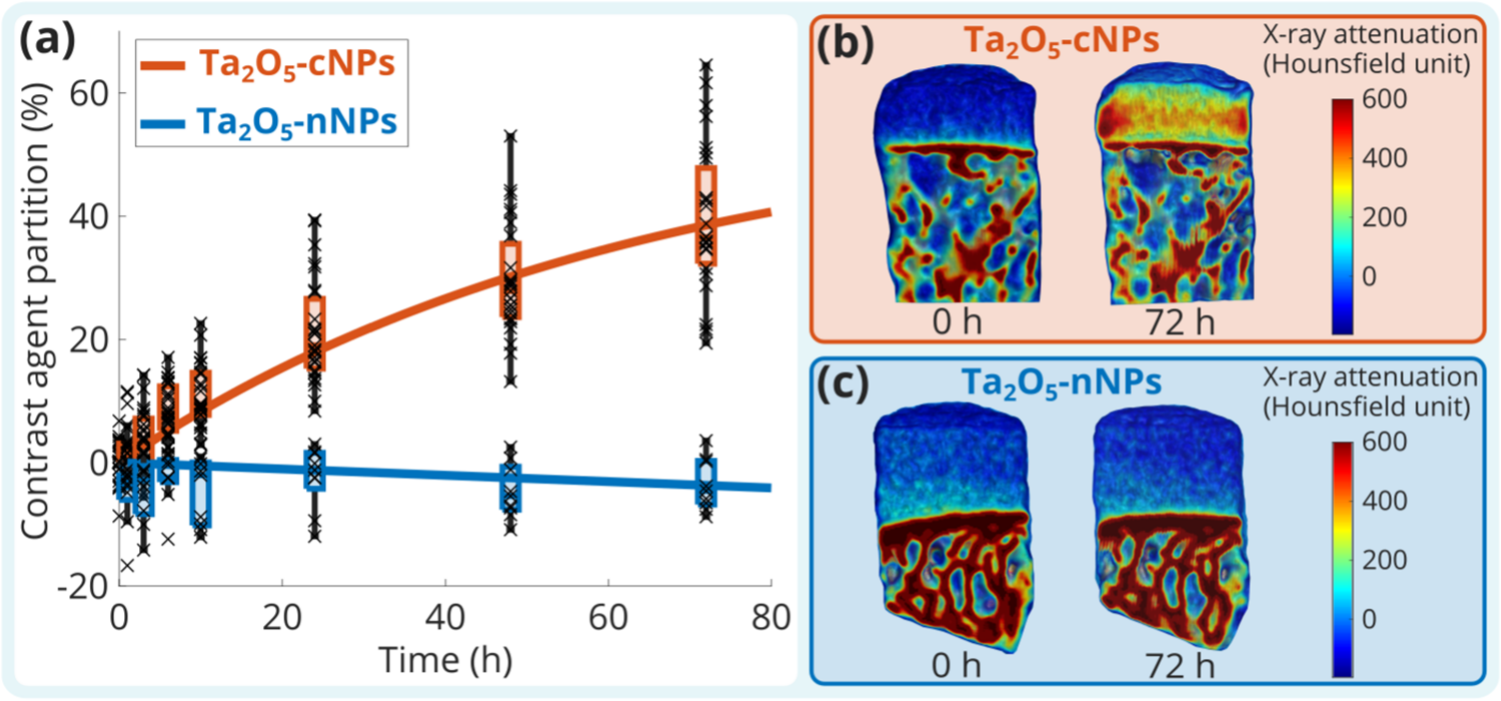
Surface charge of NPs significantly influenced their diffusion into human cadaveric articular cartilage. (a) Ta_2_O_5_-cNPs (orange) diffused into the articular cartilage, but Ta_2_O_5_-nNPs (blue) did not. The exponential equations (eq 1) for Ta_2_O_5_-cNPs and Ta_2_O_5_-nNPs were *P*(*t*) = 56.49% × [1 – exp­(−*t*/62.85 h)] and *P*(*t*) = −3139.90% × [1 – exp­(−*t*/61318.38 h)], respectively. The measured data points are presented by ×, boxplots show the deviation of the data points at different time points, and solid lines represent exponential fits using the average of fitting parameters for each NP. X-ray attenuation of samples at 0 and 72 h of diffusion in (b) Ta_2_O_5_-cNP and (c) Ta_2_O_5_-nNP baths.

Ta_2_O_5_-cNPs diffusion characteristics reflected cartilage structural and functional properties (Table 1 and Figure 3): *P*_max_ was greater in samples with higher PG content (ρ = 0.46; *p* = 0.016), and the correlation was stronger with superficial PG content (ρ = 0.58; *p* = 0.002) than with bulk PG content. *P*_max_ had a statistically significant relationship with the equilibrium modulus (ρ = 0.39; *p* = 0.047) and the stress-relaxation time constant (ρ = 0.56; *p* = 0.003) and was inversely correlated with the phase shift (ρ = −0.56; *p* = 0.003). Greater superficial PG content prolonged the time to reach contrast agent diffusion equilibrium, which was seen as a positive correlation between τ and superficial PG content (ρ = 0.54; *p* = 0.004). A similar effect was observed with a greater equilibrium modulus (ρ = 0.60; *p* = 0.001) and the stress-relaxation time constant (ρ = 0.67; *p* < 0.001). An inverse correlation was found between τ and the phase shift (ρ = −0.58; *p* = 0.002).

**Figure 3:**
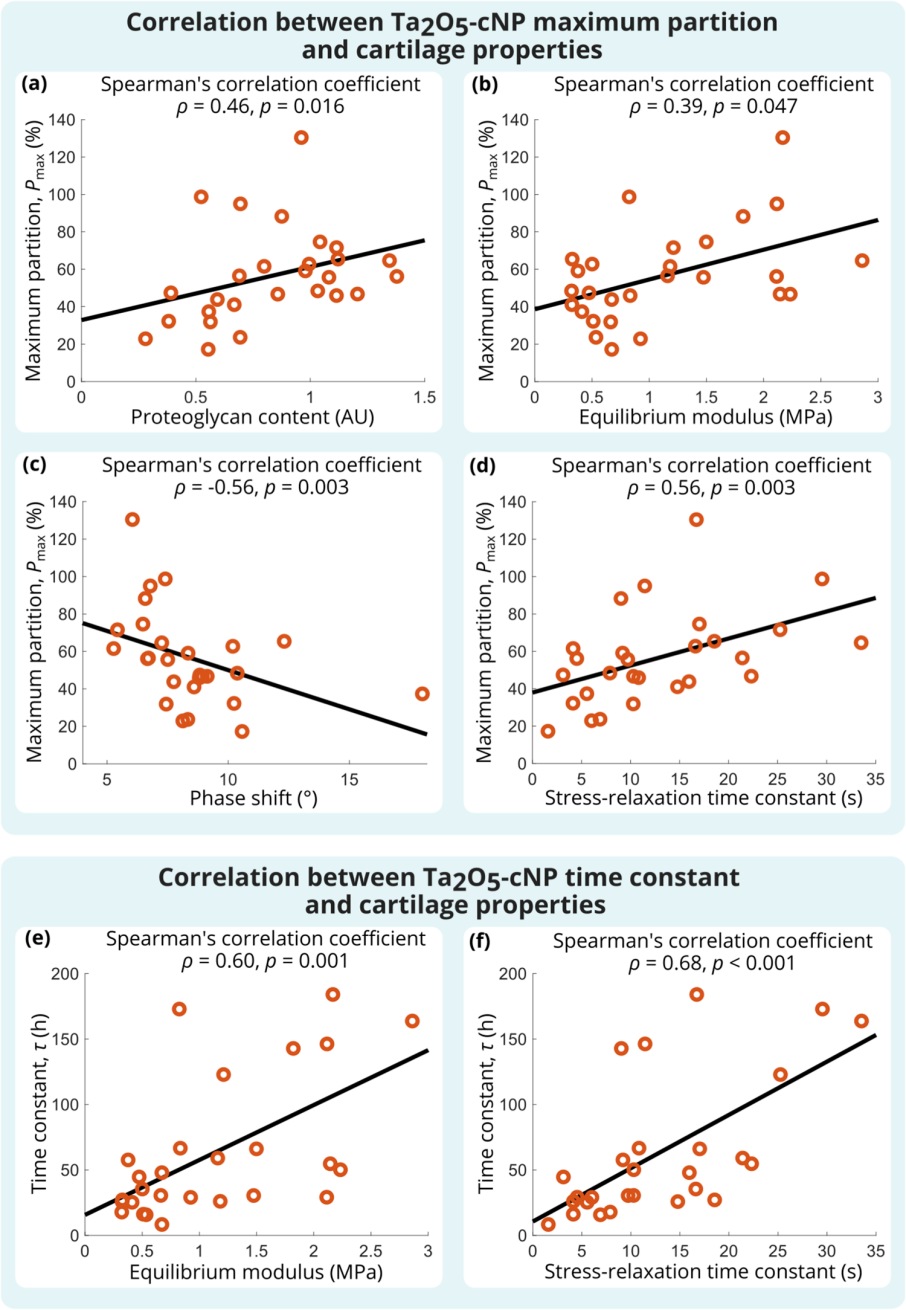
Spearman correlation coefficient between the maximum contrast agent partition of Ta_2_O_5_-cNPs and the (a) PG content, (b) equilibrium modulus, (c) phase shift, and (d) stress-relaxation time constant. Additionally, the Spearman correlation coefficient between the diffusion time constant and the (e) equilibrium modulus and (f) stress-relaxation time constant.

**Table 1:**
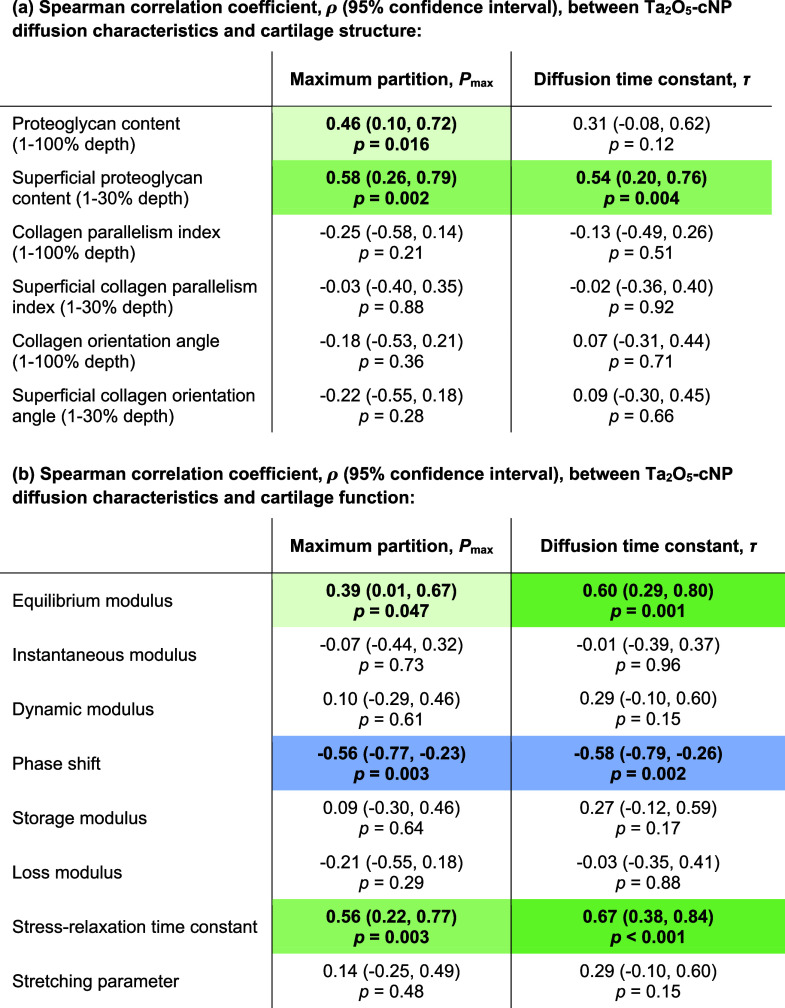
Spearman Correlation Coefficient, ρ (95% Confidence Interval), between Diffusion Characteristics (Maximum Partition, *P*_max_, and Diffusion Time Constant, τ) and the (a) Bulk and Superficial Structural Properties and (b) Functional Properties of Cartilage^a^

a The statistical significance (*p* < 0.05) is indicated by bold. The sign of the correlation coefficient is indicated by color: blue for negative values and green for positive values. The strength of the correlation coefficient is represented by color tone, with darker shades indicating stronger correlations.

The superficial lesions did not alter the diffusion of Ta_2_O_5_-nNPs because they remained excluded from the cartilage extracellular matrix [−3.04% (95% CI −5.87, −0.20) partition at 144 h on average]. Instead, Ta_2_O_5_-nNPs accumulated within the microscopic surface injuries, enabling the clear detection of the lesions (Figure 4b), which were not visible in phosphate-buffered saline (PBS; mimicking synovial fluid). The attenuation difference between cartilage and the surrounding fluid was significantly increased (3 times) with Ta_2_O_5_-nNPs compared to PBS, increasing from 77 ± 6 to 236 ± 5 HU.

**Figure 4:**
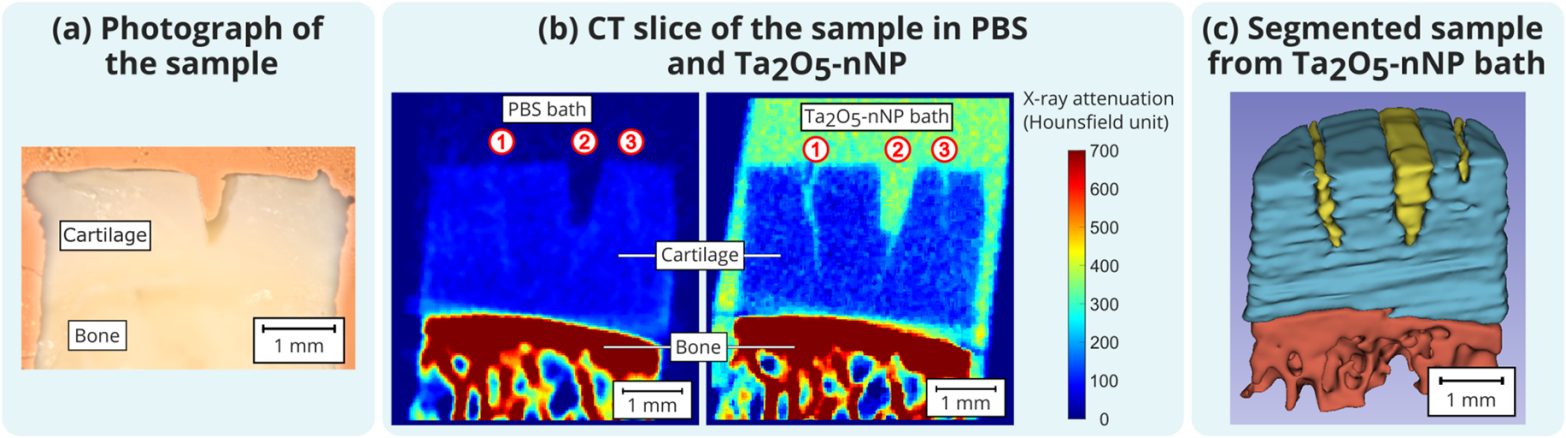
(a) Photograph of the sample showing a v-cut on the surface but invisible sharp cuts. (b) Visible surface and v-cut (number 2) in a PBS bath but obscured sharp cuts (numbers 1 and 3). In the Ta_2_O_5_-nNPs contrast agent bath, the sharp cuts become visible, and the attenuation difference between cartilage and the surrounding fluid is enhanced 3-fold. (c) Ta_2_O_5_-nNPs easing the segmentation of cartilage and visualization of the superficial lesions (cuts).

To extend the findings from the *in vitro* study involving human osteochondral samples to whole joint environments, we performed *ex vivo* large animal pilot experiments. Specifically, we injected the NPs into intact whole joints, mimicking a procedure done in the clinic. Ta_2_O_5_-cNPs were uptaken in an *ex vivo* rabbit knee joint consistent with the human *ex vivo* data (Figure 5). Similarly, we established the lesion detection capability of Ta_2_O_5_-nNPs in an *ex vivo* equine medial carpal joint (Figure 6). Healthy control cartilage appeared uniform in the CT image, while injured regions showed frequent narrow defects and broader cartilage loss in the location of linear fissures and partial erosion validated by arthroscopy. Ta_2_O_5_-nNPs enabled cartilage segmentation, although accurate delineation of individual narrow fissures was limited by resolution and contrast sensitivity.

**Figure 5:**
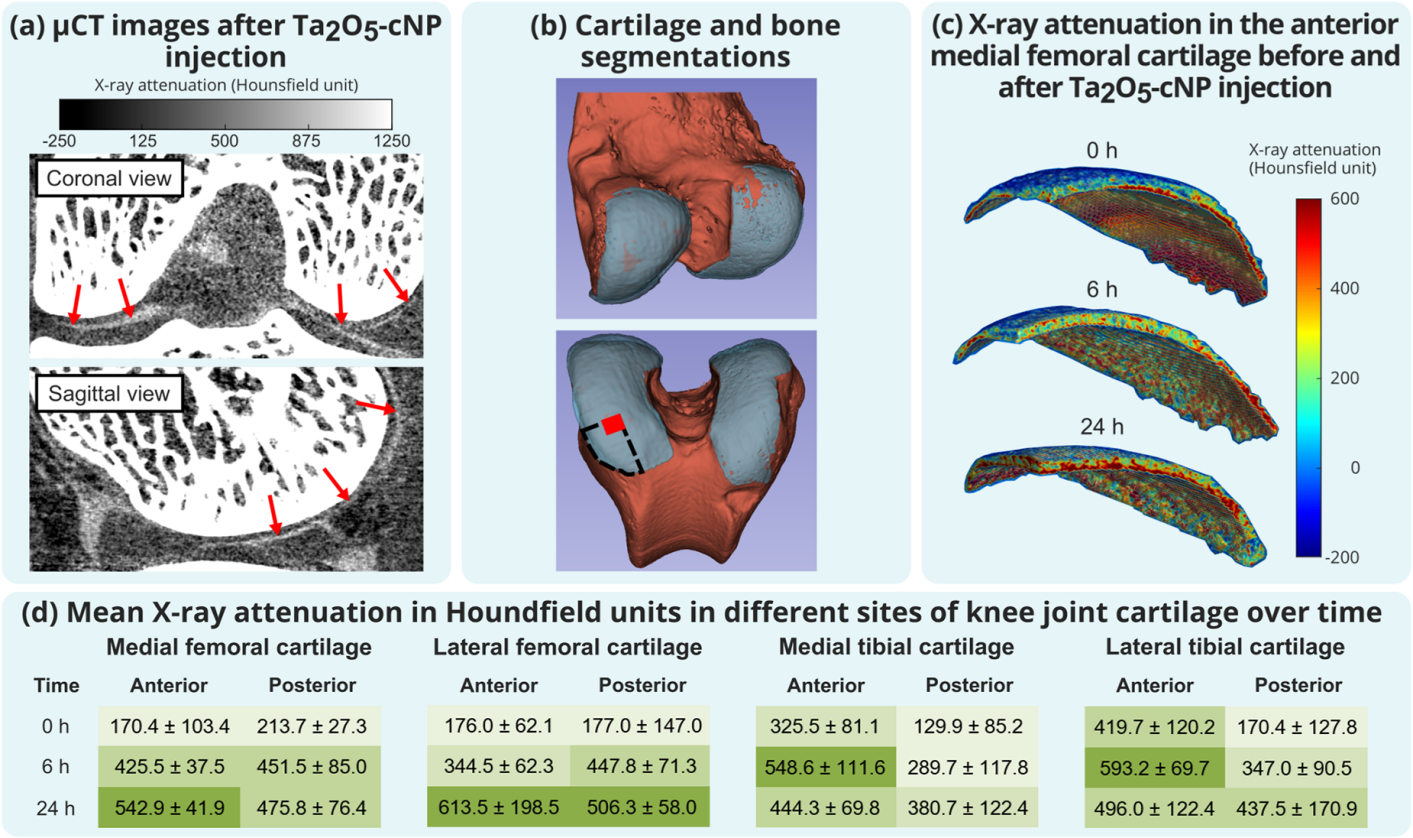
Distribution and uptake of Ta_2_O_5_-cNPs within an *ex vivo* rabbit knee joint. (a) μCT imaging performed 6 h after intra-articular injection revealed the distribution of a Ta_2_O_5_-cNP contrast agent within the joint space. (b) The contrast agent enabled segmentation of cartilage, allowing quantification of Ta_2_O_5_-cNPs uptake in all cartilage regions. (c) X-ray attenuation in the anterior medial femoral cartilage showed an increasing trend with increasing diffusion time. The red arrows point to the Ta_2_O_5_-cNP contrast agent in the joint space in part a. The black dashed line in part b indicates the location presented in part c, while the red cube highlights the analysis region within the anterior medial femoral condyle. (d) X-ray attenuation in Hounsfield units (HU, mean ± standard deviation) of different segmented anatomical sites. Different green shades represent X-ray attenuation ranges of <300 HU, 300–400 HU, 400–500 HU, and >500 HU, with darker shades reflecting higher attenuation values.

**Figure 6:**
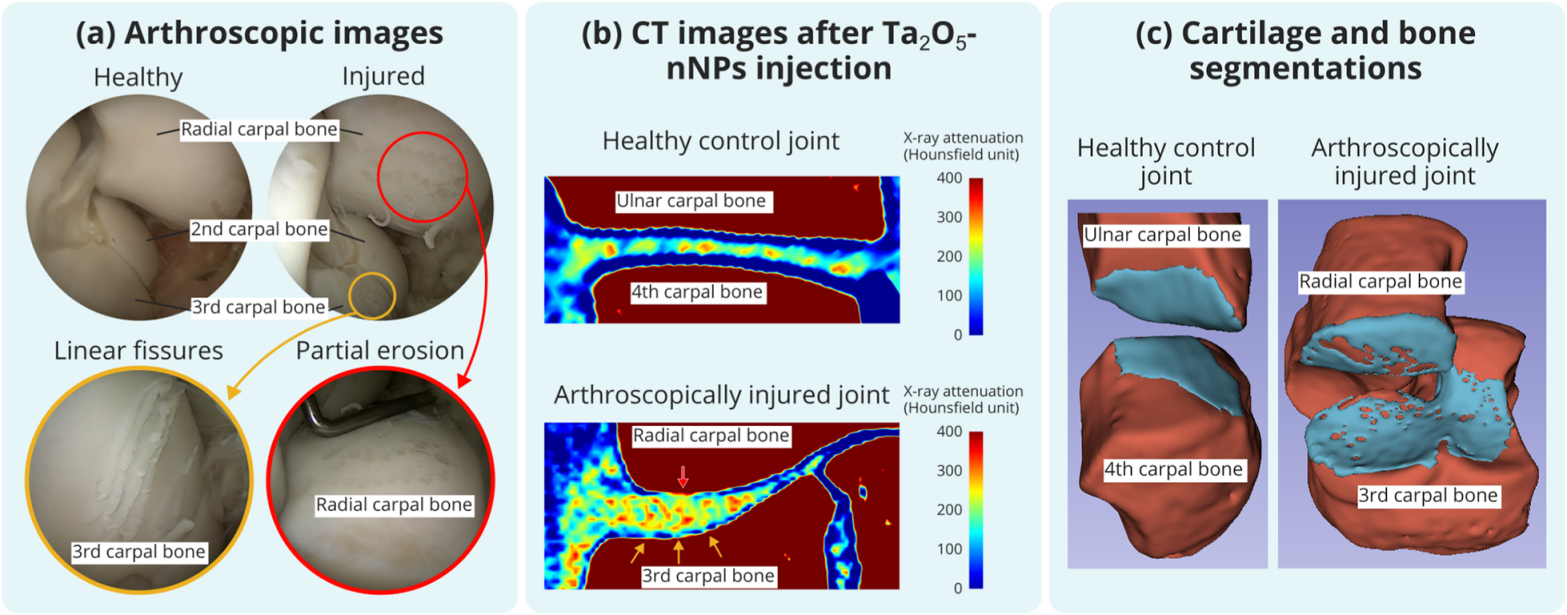
Distribution and lesion detection capability of Ta_2_O_5_-nNPs within an *ex vivo* equine medial carpal joint. (a) Arthroscopic images show cartilage surfaces of the radial and 3rd carpal bones before and after arthroscopically induced injuries. The yellow circle highlights the area with linear fissures in the cartilage, and the red circle indicates a partially eroded area. (b) A CT image of a healthy control joint between the ulnar and 4th carpal bones showed uniform and smooth cartilage surfaces, whereas injuries were evident in the injured joint. Partially eroded areas had a broader loss of cartilage, while linear fissures appeared as small narrow defects, highlighted using red and yellow arrows, respectively. The CT images were collected immediately after the Ta_2_O_5_-nNP contrast agent injection. (c) Ta_2_O_5_-nNPs enabled cartilage segmentation within the joint.

## Discussion

In this study, we introduced two comparably sized, customized ultrasmall NPs for CECT imaging of human knee cartilage: (1) Ta_2_O_5_-cNPs and (2) Ta_2_O_5_-nNPs. The cationic Ta_2_O_5_-cNPs efficiently diffuse into human cadaver cartilage, reaching 69% of their maximum partition within 72 h; however, equilibrium was not reached by the end of the measurement period. In contrast, the neutral Ta_2_O_5_-nNPs do not diffuse into the cartilage (Figure 2), despite their similar size (diameter of approximately 3 nm). This result indicates that charge–charge interactions between the negatively charged PGs and the cationic NPs govern NP uptake. These results are consistent with recent work showing that surface charge density influences the NP diffusion depth and rate in complex environments more than the NP size.^38^

After optimizing the synthetic procedure to Ta_2_O_5_-cNPs, guided by previous works,^34–36^ we obtained sufficient cytocompatibility and cationic nature by using a 1:2 volume ratio of electrically neutral PEG and positively charged trimethylammonium ligands in the NP coating. The addition of PEG in coating improves biocompatibility compared to coating only with a charged ligand and reduces NP–protein interactions, thus limiting the protein corona that could change the surface charge of NPs.^20,34,39–41^ The Ta_2_O_5_-cNP partition values within cartilage by 72 h and at the estimated equilibrium (i.e., *P*_max_) are 39% (11.8 mg/mL) and 56% (16.8 mg/mL), respectively, both of which are safely below the IC_50_ threshold for 50% cell viability, determined to be 22.43 mg/mL. More specifically, both concentrations cause less than 10% cell death (Figure S1). Accordingly, the Ta_2_O_5_-cNPs uptake is within the safety margin and sufficient to produce a high-quality image, making it suitable for CECT imaging of cartilage. In contrast, Ta_2_O_5_-nNPs do not diffuse into cartilage by 72 h, and their concentration is below the reported IC_50_ threshold of ∼20 mg/mL.^35^ Electrostatic attraction can drive cationic NPs to accumulate in cartilage at concentrations of up to ∼8 times higher than those in the bathing solution,^36,42^ which is an important biosafety consideration. In the present study, NP uptake remained moderate at levels comparable with concentrations shown to maintain high chondrocyte viability. Following intra-articular injection, contrast agents are diluted in synovial fluid and partially cleared via lymphatic drainage, reducing their effective cartilage concentration. While a 30 mg/mL dose is expected to remain within safety margins, future *in vivo* studies will quantify dilution and clearance.

*P*_max_ of Ta_2_O_5_-cNPs reflects the PG content of cartilage due to affinity between positively charged NPs and negatively charged PGs (Figure 3a).^34–36^ PGs are the main contributors to the cartilage Young’s equilibrium modulus (*E*_eq_) and a decrease in the PG content is associated with increased viscosity.^4^ Thus, the linkage between *P*_max_ and the PG content affords a positive correlation between *P*_max_ and *E*_eq_ (Figure 3b) and an inverse correlation between *P*_max_ and the phase shift (i.e., viscosity) (Figure 3c). Furthermore, the cartilage porosity increases contrast agent uptake, indicated by the positive correlation between the stress-relaxation time constant, α (i.e., porosity), and *P*_max_ (Figure 3d).

The diffusion time of Ta_2_O_5_-cNPs depends on the porosity-related parameter α (ρ = 0.68; *p* < 0.001; Figure 3f), as well as the phase shift (ρ = −0.58; *p* = 0.002; Table 1b). Such observations have not been reported previously with positively charged Ta_2_O_5_-cNPs.^36^ This finding suggests that the current NPs, with moderate cationic character achieved by balancing positive and neutral ligands in the coating, enable enhanced quantification of the tissue porosity and viscosity. In contrast, for more strongly cationic NPs, these effects are not observed, likely because excessive charge dominated the interactions, reducing the sensitivity to other tissue properties. In addition, the diffusion time of Ta_2_O_5_-cNP increases for the samples with greater superficial PG content (ρ = 0.54; *p* = 0.004; Table 1a) because it increases the amount of Ta_2_O_5_-cNPs diffusing into cartilage and decreases superficial layer diffusivity.^43^ Similarly, *E*_eq_ is indirectly related to the diffusion time (ρ = 0.60; *p* = 0.001; Figure 3e), again as a consequence of PGs. Both findings align well with our prior study utilizing Ta_2_O_5_-cNPs with a trimethylammonium coating;^36^ however, the current correlations with PG-related parameters are weaker due to the addition of neutral PEG in the NP coating. Unlike in our previous study, where Ta_2_O_5_-cNPs with only trimethylammonium coating exhibited significant relationships between Ta_2_O_5_-cNP diffusion characteristics (i.e., *P*_max_ and τ) and collagen orientation or anisotropy, no such relationships are observed here. This may be attributed to the relatively lower uptake of the current NPs, which reduces their sensitivity to these structural features.

Neutral Ta_2_O_5_-nNPs enhance the differentiation of cartilage from the surrounding fluid: the attenuation difference between cartilage and PBS (which mimics synovial fluid) is tripled by using Ta_2_O_5_-nNPs, and, thus, it improves the visibility of the cartilage surface and superficial lesions in CECT imaging, especially, the detection of sharp cuts (a width of 0.1 mm and a depth of two-thirds of the cartilage thickness; Figure 4b, cuts 1 and 3). Because dense collagen structures are known to reduce the permeability of superficial cartilage,^44^ we explored whether the well-organized superficial collagen network restricts the diffusion of neutral NPs. Damage extending into the intermediate zone of cartilage, where the permeability is higher compared to that of the superficial layer,^45^ may allow Ta_2_O_5_-nNPs to penetrate through the cut surfaces, potentially reducing contrast at the lesion site. Despite the damage, Ta_2_O_5_-nNPs do not diffuse inside the articular cartilage during an additional 72 h of follow-up, which supports our conclusion that the uptake capacity of these NPs is mainly determined by their charge. We did not study anionic Ta_2_O_5_-NPs because a prior study reported the same diffusion kinetics for neutral and anionic phosphonate-functionalized Ta_2_O_5_-NPs in murine cartilage.^34^

While *in vitro* plug studies allowed us to isolate and quantify the fundamental diffusion properties, whole-joint models are necessary to assess the translational feasibility. Therefore, we conducted *ex vivo* pilot experiments in intact joints to complement the plug studies and evaluated the diagnostic potential of Ta_2_O_5_-cNPs for cartilage imaging. The X-ray attenuation progressively increases in the rabbit knee joint following Ta_2_O_5_-cNPs administration, confirming their diffusion into cartilage tissue over time (Figure 5). Although these results demonstrate sensitivity to PG-related changes and successful uptake in joint-level geometry, further studies are needed to evaluate benchmark discrimination between healthy and osteoarthritic cartilages *in vivo*. In contrast, Ta_2_O_5_-nNPs distribute at the cartilage surface in the equine carpal joint, enabling clear visualization of the cartilage surface and facilitating lesion detection. Their ability to segment cartilage and reveal broader erosive changes and fissures highlights their utility in identifying degenerative features appearing in osteoarthritic cartilage. The segmentation of narrow fissures remains limited, but these features are visible in two-dimensional slices, enabling diagnostic interpretation. While these findings demonstrate promising imaging capabilities, full optimization of the clinical CT imaging protocols, including segmentation and spectral imaging enabled by photon-counting detector technology, remains an essential next step.

We envision that both Ta_2_O_5_-cNPs and Ta_2_O_5_-nNPs will be administered locally via intra-articular injection, as is routinely done in the clinic for OA treatments (e.g., steroids and viscosupplements).^46^ This mode of administration, as opposed to intravenous administration, ensures that the contrast agent is present in the joint, minimizes off-target toxicity to other tissues and organs, and enables the use of lower doses. Once injected, the NPs will mainly interact with the synovial fluid containing biomolecules, e.g., hyaluronic acid, albumin, globulin, and lubricin. The opposite charge of Ta_2_O_5_-cNPs relative to anionic hyaluronic acid potentially causes binding with synovial fluid, hindering its diffusion into cartilage. However, Ta_2_O_5_-cNPs are hydrophilic and cationic, and hydrophilic cationic carriers efficiently target cartilage, as reported by Vedaghavami et al., concluding that charge binding to synovial fluid does not prevent cartilage penetration.^47^ The PEGylated hydrophilic coatings preserve these charge-mediated diffusion advantages.^47^ In support of this finding, Ta_2_O_5_-cNPs efficiently diffused into cartilage when injected intra-articularly in an *in vivo* murine model.^34^ In contrast, the Ta_2_O_5_-nNPs are not electrostatically attracted to the PGs in cartilage and reside at the cartilage interface. The differential diffusion outcomes we observe arise mechanistically via electrostatic interactions with cartilage’s high negative glycosaminoglycan charge density: cationic particles benefit from enhanced partitioning and retention, whereas neutral ones do not.^48^ Consistent with this, a study controlling for size shows that the surface charge is the primary determinant of NP transport into cartilage.^49^

Once in the joint, NPs are generally thought to be cleared from the joint space via two primary mechanisms *in vivo*: small molecules (<10 kDa) diffuse through capillaries, while larger particles are removed via the lymphatic system.^50^ In a murine model, Ta_2_O_5_-cNPs accumulate transiently in the kidneys, suggesting renal excretion as a likely systemic clearance pathway.^34^ While efficient clearance helps to mitigate potential adverse effects, it also poses a challenge for maintaining sufficient intra-articular retention of contrast agents. Size and charge are key parameters for prolonging joint residence and enhancing cartilage uptake.^51,52^ Finally, tantalum oxide is regarded as biocompatible and chemically inert, and a long-term study in rats does not report significant toxicity, suggesting a low risk of harmful accumulation.^53^

## Conclusions

Taken together, these mechanistic insights strengthen the rationale for tailoring the surface charge in NP design because they directly translate to distinct imaging applications observed in this study. The cationic Ta_2_O_5_-cNPs diffuse into cartilage, providing information on the PG content and functional properties, such as stiffness, porosity, and viscosity. In contrast, the neutral Ta_2_O_5_-nNPs remain at the cartilage surface, enhancing segmentation and enabling the clear detection of superficial lesions. Complementary *ex vivo* pilot studies in intact joints further confirm the applicability of both NP variants in realistic joint geometries. These findings demonstrate that ultrasmall, surface-modified Ta_2_O_5_-NPs hold substantial potential for advancing the CECT-based characterization and diagnosis of cartilage injuries and early degeneration.

## Methods

Figure 7 illustrates an overview of the study methods, which are presented in more detail in the following sections.

**Figure 7:**
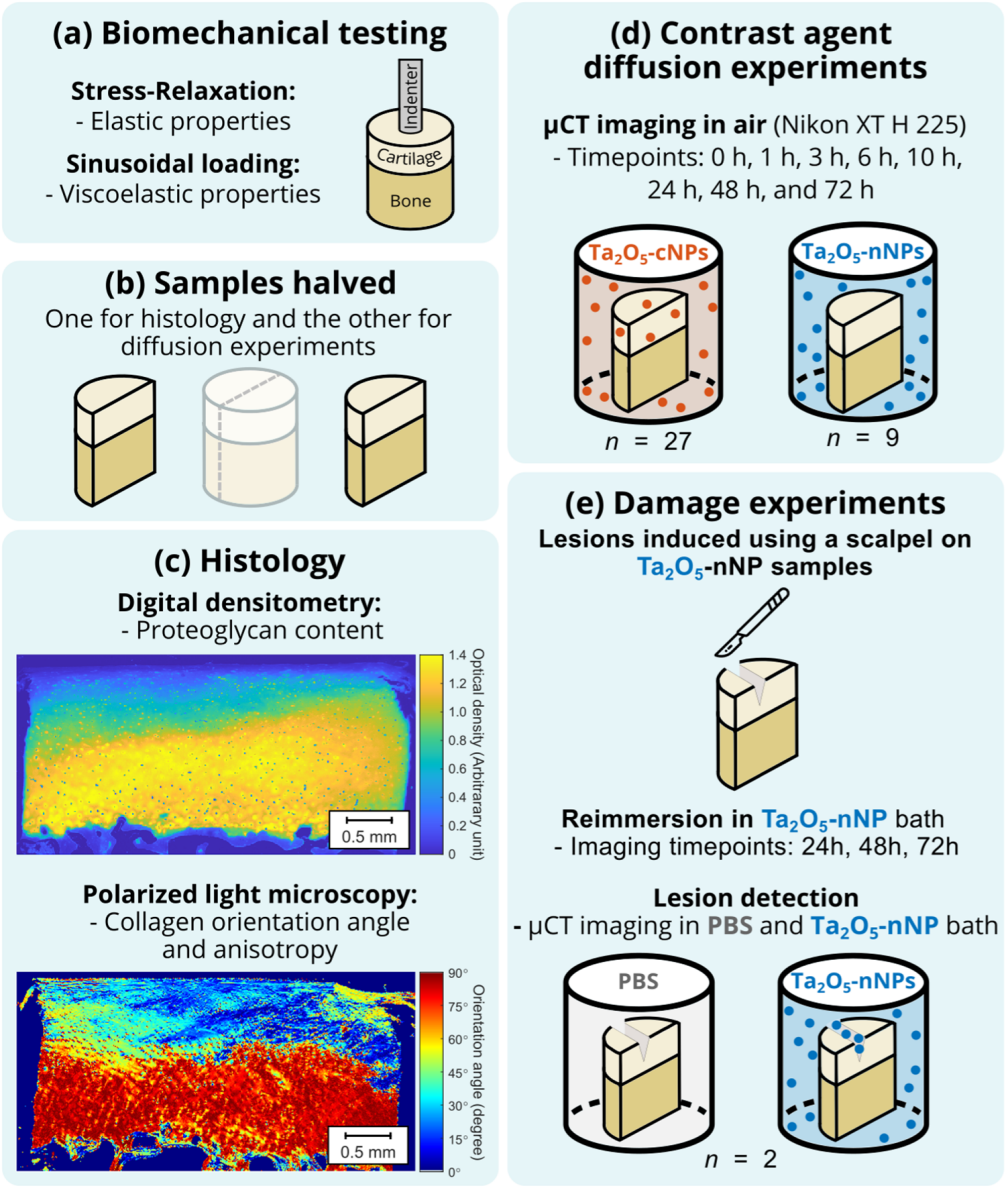
Workflow of the study: Osteochondral samples (*n* = 36; *d* = 4 mm) collected from human cadaver femurs (*N* = 7). (a) Biomechanical properties of the cartilage were determined using stress-relaxation and sinusoidal loading protocols in the indentation geometry. (b) After biomechanical testing, the samples were halved for CECT experiments, and the second half was processed for (c) histology. Digital densitometry and polarized light microscopy were used to determine the PG content and collagen network structure, respectively. (d) CT imaging was conducted using a Nikon XT H 225 μCT scanner (0 h time point), after which the samples were immersed in a Ta_2_O_5_-cNP bath (*n* = 27) or a Ta_2_O_5_-nNP bath (*n* = 9). Contrast agent diffusion into cartilage was followed by imaging samples in air after 1, 3, 6, 10, 24, 48, and 72 h of immersion. (e) Lesions were induced in the nine samples immersed in the Ta_2_O_5_-nNP bath, reimmersed in the bath, and imaged in air using μCT after 24, 48, and 72 h of additional immersion. Then two of the samples were imaged using μCT in PBS and a Ta_2_O_5_-nNP bath.

### Samples

Cylindrical osteochondral samples (*n* = 36; *d* = 4.0 mm) were extracted from the femurs of human cadaver knee joints (*N* = 7) using a 4 mm dental drill (NTI-Kahla Rotary Dental Instruments, Kahla, Germany) and stored in vials filled with isotonic phosphate-buffered saline (PBS) at −23 °C. The knee joints of human cadavers were obtained from a commercial biobank (Science Care, USA) and approved by the Research Ethics Committee of the Northern Savo Hospital District (Kuopio University Hospital, Kuopio, Finland, Decision No. 134/2015 [58/2013]).

### Biomechanical Testing

Prior to biomechanical testing, the osteochondral samples were thawed at room temperature, and the cartilage thickness was determined by tomographic imaging using a microcomputed tomography (μCT) scanner (Nikon XT H 225 μCT, Nikon Metrology Europe, Leuven, Belgium). Biomechanical properties were determined by conducting a stress-relaxation protocol^54^ and a sinusoidal loading protocol.^55^ Both protocols were conducted in indentation geometry using a custom-made mechanical testing device.^56^

The testing device consists of a displacement actuator (resolution 0.1 μm, PM1A11939, Newport, Irvine, CA, USA), a load cell (resolution 3.7 mN, Model 31, Honeywell International Inc., Charlotte, NC, USA), and a custom-made sample holder equipped with an acrylic edge and a stainless-steel bed. The osteochondral samples were fixed in the holder, perpendicular alignment of the cartilage surface to the plane-ended cylindrical indenter (*d* = 0.55 mm) was ensured using a goniometer, the holder was filled with isotonic PBS, and a prestress of 40 kPa was applied to establish full contact between the indenter and cartilage surface before the experiment.

The stress-relaxation protocol consisted of four steps (each 4% of the remaining cartilage thickness) with 600 s relaxation time after the steps, and indentation was conducted using a 100%/s ramp rate.^54^ Steps 2–4 were used to determine *E*_eq_, strain-dependent instantaneous modulus (*E*_inst_), α, and a stretching parameter (β).^13,54^ The sinusoidal loading protocol consisted of four cycles, and the peak-to-peak strain amplitude was set to 4% of the cartilage thickness and the frequency to 1 Hz.^55^ From data collected during this protocol, the dynamic modulus and phase shift were determined.

To account for the impact of the indentation geometry, the Hayes correction was applied in both testing protocols, assuming a Poisson’s ratio of 0.2 for the equilibrium modulus and 0.5 for the dynamic modulus.^57–59^ Following the biomechanical tests, the samples were halved, with half reserved for CECT experiments and the other for histology.

### Contrast Agents

Ta_2_O_5_-NPs were synthesized following a previously described protocol^34^ with modifications. Briefly, Ta_2_O_5_-NP cores were formed by hydrolyzing tantalum­(V) ethoxide (6.0 mL, Sigma-Aldrich, St. Louis, MO, USA). 1-Propanol (150 mL, Thermo Fischer Scientific, Waltham, MA, USA), isobutyric acid (2.2 mL, Sigma-Aldrich), and deuterium oxide (2.5 mL, Sigma-Aldrich) were mixed under a nitrogen atmosphere at room temperature for 16 h. Then, tantalum ethoxide was added dropwise (0.5 mL/min) and allowed to react for 16 h with stirring. After the core formation, 100 mL of 1-propanol was added dropwise (5 mL/min) to the mixture. The ligands were conjugated by adding a mixture of PEG–silane (3-[methoxy­(polyethyleneoxy)_6–9_]­propyltrimethoxysilane, Gelest Inc., Morrisville, PA, USA) and trimethylammonium (*N*-trimethoxysilylpropyl-*N*,*N*,*N*-trimethylammonium chloride, Gelest Inc.) diluted by 1-propanol (75 mL). The ligands were added dropwise (5 mL/min) at volume ratios of 1:1 (12.5 mL:12.5 mL) for Ta_2_O_5_-nNPs and 1:2 (6.25 mL:12.5 mL) for Ta_2_O_5_-cNPs. The mixture was refluxed for 4 h in 103 °C. After cooling to room temperature, ammonium hydroxide (0.1 M, 1.25 mL, Sigma-Aldrich) was added, and the reaction was stirred in room temperature for another 16 h. Weakly attached ligands were washed away under acidic conditions. First, 200 mL of deionized water was added dropwise (5 mL/min), followed by the dropwise addition of hydrochloric acid using the same rate (1.2 M, 50 mL, Sigma-Aldrich) and stirring for 48 h at 50 °C. After cooling, the solution was neutralized with sodium carbonate, followed by filtration through a 0.45 μm hydrophilic polytetrafluoroethylene filter (Whatman Cytiva, Thermo Fischer Scientific Inc.). Then the NPs were dialyzed [SnakeSkin Dialysis Tubing (3.5 kDa), Thermo Fisher Scientific Inc.] for 72 h with frequent water change in deionized water. For stability, the NPs were freeze-dried and stored as dry powder in light-protected conditions.

The hydrodynamic diameter and ζ potential of the NPs were characterized using a dynamic and electrophoretic light scattering analyzer (Brookhaven Nanobrook Omni Particle Sizer and Zeta Potential Analyzer, Brookhaven Instruments, Holtsville, NY). The core size of the NPs was determined using ultrahigh-resolution analytical scanning transmission electron microscopy (UHR-STEM, Model JEM-ARM200F, Jeol Ltd., Tokyo, Japan; resolution = 33 pm) operating at 200 kV. UHR-STEM samples were prepared by drying 5 μL of a 1.0 mg/mL NP solution in nanopure water on the grid (Lacey Formvar/Carbon, 300 mesh, Copper, Ted Pella Inc., Redding, CA, USA). *ImageJ* software was used to measure the projected core diameter of 100 NPs of each type from dark-field UHR-STEM images. Fourier transform infrared spectroscopy (FTIR; Thermo Nicolet iS50, Thermo Fisher Scientific Inc., Waltham, MA, USA) was used to verify ligand conjugation to the particle surface. The cytotoxicity of Ta_2_O_5_-cNPs was assessed by culturing human chondrocytes (NHAC-kn, Lonza, Walkersville, MD, USA) with various concentrations of Ta_2_O_5_-cNPs in Alamar Blue assay.

Before use, Ta_2_O_5_-NPs were reconstituted in a physiological buffer (pH 7.4) to maintain colloidal stability. Ta_2_O_5_-nNPs and Ta_2_O_5_-cNPs in powder were dissolved in PBS containing protease inhibitors [5 mM ethylenediaminetetraacetic acid disodium salt dihydrate (EDTA; VWR International, Radnor, PA, USA) and 5 mM benzamidine hydrochloride hydrate (Sigma-Aldrich Co., St. Louis, MO, USA)]. The bath concentration of Ta_2_O_5_-nNPs and Ta_2_O_5_-cNPs was set to 30 mg/mL to ensure sufficient attenuation based on a previous study.^36^ The pH and osmolality were adjusted to 7.4 and 400 mOsm/kg, respectively, to mimic the pH and ionic strength of the synovial fluid.^60^ Suspensions prepared for experiments are stored short-term at 4 °C and gently resuspended if used after several days. The contrast agent bath volume was fixed to 2.50 mL per sample, i.e., 200 times the estimated cartilage volume, to minimize dilution of the bath due to diffusion.

### CECT Imaging of Cartilage

#### Diffusion Experiments

To allow the contrast agent to diffuse solely through the articulating surface, we sealed the edges and bottom of the osteochondral samples using cyanoacrylate (Super Glue, Loctite, Henkel AG, Düsseldorf, Germany). Subsets of the samples were immersed in the Ta_2_O_5_-nNP (*n* = 9) and Ta_2_O_5_-cNP (*n* = 27) baths at 37 °C for 72 h, with continuous shaking throughout the immersion period. Imaging was conducted in air at various time points (0, 1, 3, 6, 10, 24, 48, and 72 h) using a Nikon XT H 225 μCT scanner with a tube voltage of 150 kVp and 0.5 mm copper filtering. A total of 1440 frames were collected around the samples (360°) within the 6 min scan, and the imaging resulted in 40 × 40 × 40 μm^3^ voxel size.

To study how defects in the well-organized superficial zone of intact cartilage would alter the diffusion, two sharp cuts and one v-cut were induced to allow Ta_2_O_5_-nNPs to diffuse directly to the cartilage intermediate zone.^43^ The sharp cuts were done by pressing a scalpel until two-thirds depth of cartilage (0.1 mm wide) and the v-cut by removing a piece of cartilage until the same depth (width at surface approximately 0.5 mm). After the cutting, samples we reimmersed into the Ta_2_O_5_-nNP contrast agent bath for an additional 72 h and imaged in air using μCT after 24, 48, and 72 h.

#### Segmentation Experiments

μCT imaging of cartilage was conducted also in aqueous solutions (PBS and Ta_2_O_5_-nNP contrast agent) to mimic the physiological conditions of the synovial space. Two samples with superficial lesions were immersed separately into the Ta_2_O_5_-nNP contrast agent and the other in PBS, and they were subsequently imaged using μCT. This was repeated with the immersion solution reversed. The tube voltage and filtering were similar to diffusion experiments. However, the scan time was longer (53 min), and more frames were recorded with a higher resolution (4476 frames, 13 × 13 × 13 μm^3^).

### Diffusion Analysis

Contrast agent uptake was determined using a custom-made MATLAB (R2022b, MathWorks Inc., Natick, MA, USA) script as follows. First, the cartilage surface was aligned horizontally, rotating the image in the *x* and *y* directions. Then, the cartilage tissue was distinguished from air and bone, based on X-ray attenuation thresholds. A region-of-interest (ROI) with a diameter of 1.2 mm was selected from the middle of the sample for each slice, and the average attenuation was calculated. Then a depthwise attenuation profile was generated by linearly interpolating the results from all depths to 100 points. Native cartilage attenuation was removed from the profiles of immersion time points by subtracting the 0 h attenuation profile (imaged before immersion). Finally, contrast agent partitions were established by dividing the contrast agent attenuation profiles by the attenuation in the contrast agent bath.

Time-dependent diffusion was studied by conducting an exponential fit to the time-dependent partition data according to the equation

1 $$P\left(t\right)=P_{\max}\times \left[1-\exp\left(-t/\tau \right)\right]$$

where *P*_max_ is the maximum partition, *t* is the diffusion time, and τ is diffusion time constant, i.e., the time needed to reach 63.2% of *P*_max_.^12^

### Histology

Digital densitometry and polarized light microscopy were employed to assess the PG content and collagen network organization of cartilage from histological sections. Bulk properties were defined as the average across the entire cartilage thickness (1–100%) and superficial properties as the average from the top 1–30% of the cartilage thickness. The histological sections were prepared from the halves of the samples that were not used for the CECT experiments. The halves were fixed in formalin and decalcified in EDTA for 2 weeks. Then the halves were dehydrated in a series of graded alcohols and embedded in paraffin, after which 3 and 5 μm sections were sliced perpendicularly to the cartilage surface for digital densitometry and polarized light microscopy, respectively, using a microtome.^4,61^

For digital densitometry, 3 μm sections were stained with 0.5% Safranin-O, which binds stoichiometrically to PGs. When imaged using digital densitometry, Safranin-O absorbs light, allowing for assessment of the location and quantity of PGs. The digital densitometry setup consists of a light microscope (Nikon Microphot-FXA, Nikon Co., Tokyo, Japan), a CCD camera (Hamamatsu ORCA-ER, Hamamatsu Photonics, Hamamatsu, Japan; pixel size = 3.09 μm × 3.09 μm), and a monochromator (λ = 492 ± 5 nm). Calibration filters (optical densities of 0.0, 0.15, 0.3, 0.6, 1.3, 1.6, 2.0, 2.3, 2.6, and 3.0) were imaged, and linear conversion was used to convert histological images to optical densities. Three sections of each sample were imaged, the cartilage surface was oriented horizontally, pixels at the same depth were averaged for each section to generate a depthwise profile, and the depthwise profiles from the three sections were averaged.^62,63^

For polarized light microscopy, 5 μm sections were utilized, and a light microscope (Leitz Ortholux II POL, Leitz Wetzlar, Wetzlar, Germany) equipped with a monochromatic light source (λ = 630 ± 5 nm; Edmund Optics Inc., Barrington, NJ, USA), two crossed polarizers (Techspec Optics XP42-200, Edmund Optics, Barrington, NJ, USA), and a monochrome camera (BF-U3-88S6M-C FLIR Blackfly, FLIR System Inc., Wilsonville, OR, USA; pixel size = 3.5 μm × 3.5 μm) was used to determine the collagen fiber network organization and parallelism.^64,65^ The cartilage surface was oriented horizontally, one section was imaged, and a depthwise profile was generated by averaging pixels at the same depth.^4,64^

### *Ex Vivo* Experiments

*Ex vivo* experiments were conducted to investigate the applicability of both NP types in the joint geometry. Ta_2_O_5_-cNPs (30 mg/mL) were injected intra-articularly *ex vivo* into a rabbit knee joint and imaged using μCT (vivaCT80, Scanco Medical AG, Brüttisellen, Switzerland) with an isotropic voxel size of 35 μm^3^ (70 kVp, 0.5 mm Al filter) before and after 6 and 24 h of immersion. Ethical permission was not required because the animals were slaughtered for food purposes. Bone structures (femur and tibia) were segmented at each time point based on the attenuation threshold, while cartilage was manually segmented using CT images at the 6 h time point. Segmentations were performed using a 3D Slicer (version 5.8.1).^66^ CT images at different time points were registered based on the bone segmentations using the automated Prostate MRI-US Contour Propagation extension in the 3D Slicer. X-ray attenuation was analyzed from registered images within a 3.5 mm × 3.5 mm ROI in the anterior and posterior regions of the medial and lateral femoral cartilage.

The capacity of the Ta_2_O_5_-nNPs to highlight cartilage lesions was studied by using an equine medial carpal joint. The equine samples were collected from horses that were euthanized for other medical reasons and whose tissues were donated for research. Cartilage defects were created arthroscopically between the radial carpal bone and the third carpal bone. A sharp trocar was used to create linear fissures, while a motorized resector was used to create partial-thickness erosion. The contrast agent (30 mg/mL) was injected intra-articularly and imaged using a clinical whole-body CT scanner (135 kVp, Al and Cu filter, Cannon Aquilion Exceed LB, Canon Medical Systems Corp., Japan). A 3D Slicer was used for manual cartilage segmentation and thresholding-based bone segmentation.

### Statistical Analyses

Statistical analyses were conducted using MATLAB. Means are reported alongside their 95% CI. The Shapiro–Wilk test indicated that the data were not normally distributed (*p* < 0.05), and the limited number of samples did not allow for comprehensive statistical models. Thus, Spearman’s rank correlation test was used to examine the relationship between the contrast agent diffusion characteristics and the cartilage structure and function.

## Supplementary Material



## Notes

Cytotoxicity of Ta_2_O_5_-cNPs on human chondrocytes (Figure S1) (PDF)
