# Supplementary material for: Ultrasmall Surface-Charge-Modified Tantalum Oxide Nanoparticles for the Assessment of Articular Cartilage Using Contrast-Enhanced Computed Tomography
Source: ACS Nano. 2026 Jan 14;20(3):2717–29. doi: 10.1021/acsnano.5c15722 (PMC12854743; doi:10.1021/acsnano.5c15722)
Supplement: Supplementary file 1 [file nn5c15722_si_001.pdf]

SUPPORTING INFORMATION

Ultrasmall Surface-Charge-Modified Tantalum Oxide Nanoparticles for Assessment of Articular Cartilage Using Contrast-Enhanced Computed Tomography

Jiri Jäntti[1,2], Juuso Tuppurainen[1,2], Anisha Joenathan[3], Henri P.P. Leskinen[1,4], Annunzia M. Cagnoni[5,6,7], Heta Mertano[1,2], Milka Poimala[1,2], Ervin Nippolainen[1], Isaac O. Afara[1,8], Juuso T.J. Honkanen[9], Hanna Matikka[10], Juha Töyräs[1,8,11], Brian D. Snyder[12], Kathryn S. Stok[5], Brad B. Nelson[13], Mark W. Grinstaff[3,14], Janne T.A. Mäkelä[1]

[1]Department of Technical Physics, University of Eastern Finland, Kuopio, Finland; [2]Diagnostic Imaging Center, Kuopio University Hospital, Wellbeing Services County of North Savo, Kuopio, Finland; [3]Division of Materials Science, Boston University, Boston, USA, Boston University, Boston, USA; [4]Medical Physics, Mikkeli Central Hospital, South Savo Wellbeing Services County, Mikkeli, Finland; [5]Department of Biomedical Engineering, The University of Melbourne, Australia; [6]Rehabilitation Sciences Institute, University of Toronto, Canada; [7]Joint Department of Medical Imaging, University Health Network, Canada; [8]School of Electrical Engineering and Computer Science, The University of Queensland, Brisbane, Australia; [9]Center of Oncology, Kuopio University Hospital, Wellbeing Services County of North Savo, Kuopio, Finland; [10]ICMT and development services, Kuopio University Hospital, Wellbeing Services County of North Savo, Kuopio, Finland; [11]Science Service Center, Kuopio University Hospital, Wellbeing Services County of North Savo, Kuopio, Finland; [12]Department of Orthopedic Surgery, Boston Children's Hospital, Boston, USA; [13]Orthopaedic Research Center, C. Wayne McIlwraith Translational Medicine Institute, Colorado State University, Fort Collins, Colorado, USA; [14]Departments of Biomedical Engineering and Chemistry, Boston University, Boston, USA

*Corresponding author: Jiri Jäntti, E-mail: jiri.jantti@uef.fi

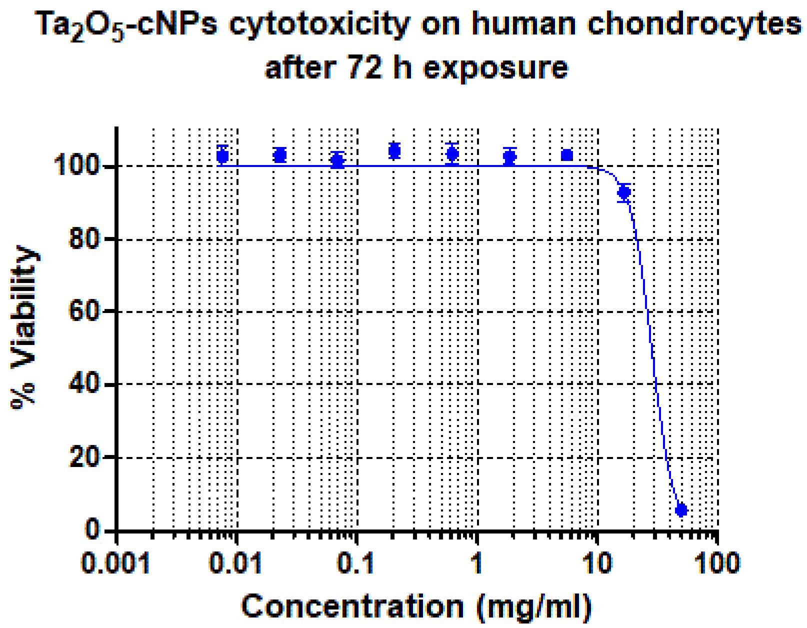

Figure S1: Dose-dependent cytotoxicity of Ta_2O_5-cNPs on human chondrocytes after 72-hour exposure: Viability remains high at low concentrations (<10 mg/ml) and corresponding half maximal inhibitory concentration (IC_{50}) value was 22.42 mg/ml.
